# Organizational Determinants of Interprofessional Collaboration in Integrative Health Care: Systematic Review of Qualitative Studies

**DOI:** 10.1371/journal.pone.0050022

**Published:** 2012-11-29

**Authors:** Vincent C. H. Chung, Polly H. X. Ma, Lau Chun Hong, Sian M. Griffiths

**Affiliations:** Jockey Club School of Public Health and Primary Care, Chinese University of Hong Kong, Hong Kong, Special Administrative Region, People's Republic of China; Tehran University of Medical Sciences, Iran (Islamic Republic of)

## Abstract

**Context:**

Inteprofessional collaboration (IPC) between biomedically trained doctors (BMD) and traditional, complementary and alternative medicine practitioners (TCAMP) is an essential element in the development of successful integrative healthcare (IHC) services. This systematic review aims to identify organizational strategies that would facilitate this process.

**Methods:**

We searched 4 international databases for qualitative studies on the theme of BMD-TCAMP IPC, supplemented with a purposive search of 31 health services and TCAM journals. Methodological quality of included studies was assessed using published checklist. Results of each included study were synthesized using a framework approach, with reference to the *Structuration Model of Collaboration*.

**Findings:**

Thirty-seven studies of acceptable quality were included. The main driver for developing integrative healthcare was the demand for holistic care from patients. Integration can best be led by those trained in both paradigms. Bridge-building activities, positive promotion of partnership and co-location of practices are also beneficial for creating bonding between team members. In order to empower the participation of TCAMP, the perceived power differentials need to be reduced. Also, resources should be committed to supporting team building, collaborative initiatives and greater patient access. Leadership and funding from central authorities are needed to promote the use of condition-specific referral protocols and shared electronic health records. More mature IHC programs usually formalize their evaluation process around outcomes that are recognized both by BMD and TCAMP.

**Conclusions:**

The major themes emerging from our review suggest that successful collaborative relationships between BMD and TCAMP are similar to those between other health professionals, and interventions which improve the effectiveness of joint working in other healthcare teams with may well be transferable to promote better partnership between the paradigms. However, striking a balance between the different practices and preserving the epistemological stance of TCAM will remain the greatest challenge in successful integration.

## Introduction

### Integrating Allopathic Biomedicine and Traditional, Complementary and Alternative Medicine

The use of traditional, complementary and alternative medicine (TCAM) and allopathic biomedicine (BM) are gaining popularity amongst patients, especially those with chronic non-communicable diseases. This poses a challenge for continuity and coordination of care as patients may consult both TCAM practitioners (TCAMP) and BM doctors (BMD) separately for their illnesses without notifying respective clinicians. Better organizational integration between TCAM and BM is a possible way of addressing this challenge, and the term “integrative healthcare” (IHC) is being widely used in policy documents and literature to describe a positive relationship between the two paradigms [1]. However, there is no consensus on the definition of integration, or at which level of the health system the IHC concept should be applied [2]. Johnson *et al.* suggested that the interprofessional collaboration (IPC) between BMD and TCAMP in health systems could be regarded as a core characteristic in IHC initiatives [3]. In healthcare, IPC could be defined as “*an interpersonal process characterized by healthcare professionals from multiple disciplines with shared objective, decision making, responsibility, and power working together to solve patient care problems; the process is best attained through an interprofessional education that promotes an atmosphere of mutual trust and respect, effective and open communication, and awareness and acceptance of the roles, skills and responsibility of the participating disciplines*” [4].

To realize the potential of IPC concepts in fostering IHC development, theoretical insights should be translated into practical organizational and management strategies by policy makers and in managers' toolbox [5]. Boon *et al.* suggest that sharing of governance, payment, care protocols and structure between the two services improves integration [6], whilst Templemen *et al.* suggest that while IPC may take different forms depending on the features of different health systems, their development process shares certain keys to success. These include support and commitment from senior management, adequate resources and funding, availability of patient record sharing systems as well as jointly agreed referral guidelines [7]. Their findings are consistent with existing emphasis on using organizational strategies in fostering IPC [8], [9], [10], [11]. This work represents initial attempts in translating IPC concepts into IHC practice, but more comprehensive guidance is needed.

In view of this gap, we conducted a systematic review to identify organizational strategies that would facilitate IPC between BMD and TCAMP. Starting with an introduction to the concepts and theories of IPC, we describe our methodology before discussing a synthesis of how organizational measures may foster IPC between BMD and TCAMP. We conclude with a discussion of how IPC experience amongst other healthcare professions may provide insights for bridging between BMD and TCAMP.

## Methods

### Ethics Statement

This is a systematic review of published papers and hence ethics approval was not sought.

### Search Strategies and Study Selection

We searched four international electronic databases (MEDLINE, EMBASE, AMED, and CINAHL) from their inception until March 2011 for qualitative studies on IPC between BMD and TCAMP. The search keywords of each database are shown in Appendix S1. In addition, we performed a purposive electronic search for TCAM qualitative studies in 16 health services journals and 15 TCAM journals. For TCAM journals, we have chosen those which ranked in the top 20 within the “Integrative & Complementary Medicine” category of the 2009 and 2010 Journal Citation Reports. To ensure comprehensiveness, we also purposefully sampled TCAM and health services journals not indexed in that category, but are likely to contain related qualitative studies.

The search strategies are listed in Appendix S2. To be eligible for inclusion in this review, the studies must satisfy all of the following criteria: (i) that they employed either a qualitative methodology including case study, focus group, interviews or ethnographic observation techniques, or a mixed methodology with a clear qualitative component; (ii) that they explicitly aimed at investigating how BMD and TCAMP collaborate; and (iii) that they performed original data collection from BMD, TCAMP or managers who were directly involved in providing or managing IHC services. Meanwhile, studies with one or more of the following features are excluded: (i) they were review articles; and (ii) they studied BMD, TCAMP or managers' general views on TCAM outside the context of IHC service provision. Two reviewers (VC and PM) independently screened the titles and abstracts to assess their eligibility. Articles that were clearly incompatible with the three inclusion criteria; or found to satisfy the two exclusion criteria, were excluded at this stage. For the remaining citations, we confirmed their eligibility by examining their full texts, and judgments were made after detailed examination of the whole article. The final decision on inclusion was made by consensus adjudication between all authors.

### Assessment of Methodological Quality

A recent systematic review of critical appraisal tools for qualitative studies has located eight instruments [12]. Amongst the eight, seven did not provide comprehensive explanation on how the appraisal criteria were developed, or clear guidance on how the instrument should be applied. It was concluded that only one instrument [13] provided a clear explanation and guidance on its application, and thus it is chosen as a tool for methodological quality assessment in the current review. By using this 12-item tool, two researchers (VC and PM) independently assessed the methodological quality of all included studies and they resolved disagreement by discussion. Given that there was no widely accepted, empirically based method for excluding qualitative studies from systematic reviews, the studies were not excluded or weighted on the basis of methodological quality [14], [15].

### Data Extraction and Analysis

For each included study, the texts under the headings of “results” or “findings” were extracted and subjected to independent analysis by two investigators (VC and PM). Given the existence of a prominent theoretical framework on IPC, analysis of qualitative findings followed a framework approach [16]. It is a commonly used; matrix based analytical technique in qualitative synthesis, in which themes from theoretical framework are identified *a priori* as coding categories at the beginning of analysis. These themes are then combined with emerging new concepts during the analysis process [17]. Based on organizational sociology, the *Structuration Model of Collaboration* stipulates that IPC is influenced by two major organizational factors: Governance and Formalization. The degree of Governance success is operationalized by four indicators: *Centrality*; *Leadership*; *Support for Innovation*; and *Connectivity*. On the other hand, Formalization, which clarifies expectations and responsibilities, can be operationalized by two indicators: *Formalization Tools and Information Exchange* [11]. Detailed elaboration on each indicator can be found in Table 1. In this review, these six organizational-level indicators were adopted as the *a priori* framework for line-by-line coding of the extracted results. Two investigators compared their coding results, and reached consensus upon a common organization of extracted data. A final synthesis was then generated after a critical discussion between all of the authors [18].

**Table 1:** Organizational Dimensions of the Structuration Model of Collaboration.∧

Organizational dimensions	indicators	description
Governance	Centrality	• Centrality refers to the existence of a clear and explicit direction towards collaboration between professions.
		• Central directives are essential strategic and political tools that help to materialize the implementation of collaborative processes and structures.
	Leadership	• Frontline leadership is essential for the success of IPC. The power differential between partners should be minimized.
		• Power should not be concentrated in the hands of a single partner; all partners must be able to have their opinions heard and to participate in decision-making.
	Support for innovation	• Collaboration often involves dividing responsibilities differently between professionals and between institutions. It necessarily entails innovations in clinical practices and in the sharing of responsibilities between partners.
		• Interprofessional learning and expert support is essential for implementing these innovations.
	Connectivity	• Strong connectivity allows for rapid and continuous adjustments to problems arising from coordination.
		• It takes the form of information and feedback systems, committees, etc.
Formalization	Formalization tools	• They are the means of clarifying the various partners' responsibilities and negotiating how the responsibilities are shared.
		• For professionals, it is important to know what is expected of them and what they can expect of others.
	Information exchange	• Refers to the existence and appropriate use of an information infrastructure that allow for rapid and complete exchanges of information between professionals.
		• Feedback provides professionals with the information they need to follow up with patients as well as to evaluate their partners on the basis of the quality of the written exchanges and feedback.

∧ Adapted from D'Amour et al., ***BMC Health Services Research*** 2008, 8:188.

## Results

### Study Selection and Characteristics

**Figure 1:**
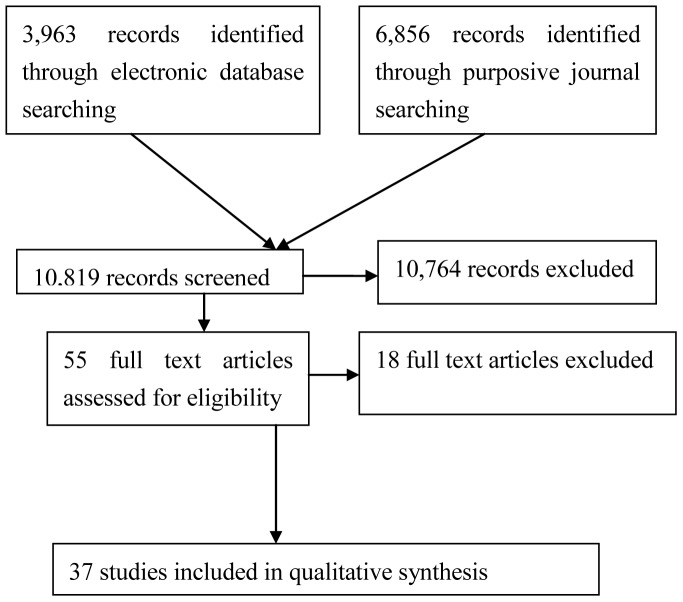
Flow of literature search and selection.

We screened 10,819 abstracts and 55 full texts were retrieved for further assessment. With the application of exclusion criteria, 18 papers were considered ineligible. Finally, 37 qualitative studies were included, and the literature flow is shown in Figure 1. Table S1 provides details of the characteristics of all included studies. Geographically, 11 studies originated from the UK and 11 studies were conducted in Canada. Four were conducted in Israel, three in Denmark, and two in Australia and USA, respectively. Sweden and South Africa each contributed one study. Two studies were of a multinational nature. One study collected data in both Israel and the USA, and the other was conducted in Canada and the USA. In terms of participants, 29 studies interviewed both BMD and TCAMP, six studies interviewed BMD only, and one collected data from TCAMP exclusively. Twelve papers mentioned interviews with managers, and respectively 19 and six studies collected data from allied health professionals and patients. Table S2 describes the methodological quality of the included studies. Nineteen papers failed to clearly elaborate the background of the issue (item 1); 37 studies did not describe the researchers' role (item 3); three studies did not illustrate the data collection process (item 6); 17 studies did not present the data analysis clearly (item 7); one study did not describe the result clearly (item 8); six papers failed to cite the respondents' direct quotations (item 9); three studies did not interpret the result in the discussion part (item 10); 22 papers did not mention the limitations of the study (item 11); and 29 papers did not propose avenues for further research (item 12). All included studies satisfied the requirements of the remaining items. A summary of our synthesis is presented in Table S3.

### Governance

Successful governance of integration often requires innovations in service organizations with clear direction and support for professionals, particularly in formulating treatment plans that combine both BM and TCAM. Governance capacity can be reflected by four indicators: *Centrality*; *Leadership*; *Support for Innovation*; and *Connectivity* [11]. We synthesized findings related to governance using these four indicators.

#### Centrality

Centrality refers to the strategic and political roles of the central authority in creating “clear and explicit direction” for guiding IPC, particularly in fostering consensus amongst organizations [11]. For developing IHC, external opposition from regulatory bodies [19], as well as internal resistance from members of top management are the first and foremost barriers to be overcome [20], [21]. Motivation and incentives for central authorities to overcome these obstacles include the need to respond to patients' demands for holistic care and the need to fill the effectiveness gaps of BM [22]–[26]. Both of these appear to increase support for integration amongst policy makers.

Active “bottom up” requests from patients for integration puts the inclusion of TCAM onto the management agenda of BM institutions [27]–[30]. Proposing the use of TCAM for managing conditions for which BM treatment has little to offer (*i.e.*, filling BM's effectiveness gaps) appeared to receive less resistance [20], [22], [31]–[36]. Gaining legitimatization by highlighting limitations of BM may promote the adoption of TCAM treatments as a last resort when all BM treatments are exhausted [19], [37], [38]. At times TCAM is regarded as a first-line treatment for “difficult to treat” diseases given its inexpensive and non-invasive nature [31]. BMD may also regard TCAM as being useful for reducing their workload when it serves as a “triage for difficult patients” [32]. The scope of effectiveness gaps also includes preventive and empathetical aspects of care (*i.e.*, treating illnesses, not diseases) that are often impractical to implement during a short BM consultation [27], [31], [35], [39], [40]. Enhancing the patient-centeredness of BM services represents another important incentive for organizational and management support for developing integrative services [37], [41], although some BMD believe that conventional care alone is holistic enough for promoting wellness [23].

Whereas the potential financial benefits to BM institutions and practitioners form the business case for providing IHC services [20], [38], [42], [43], securing funding for launching and sustaining IHC services is a major challenge for the central authorities before such gain is harvested [19], [28], [37]. Sufficient funding is not only essential for recruiting experienced, qualified TCAMP [44], but it also carries strong implications for TCAMP's sense of belonging within predominantly BM institutions as well as effecting the intention to work collaboratively with BMD [19], [35], [45]. When TCAMP are employed on a part-time fee-for-service basis, they are often reluctant to perform tasks that are unrelated to direct patient care, such as important communication and team-building activities like case conferences because many TCAMP may feel that they are not remunerated for such activities [43], [46], [47]. In some integrative settings, BMD and TCAMP are allowed to compete for patients and this means that IPC is not considered to be viable [34].

On the other hand, financial support for patients to receive TCAM treatment is also critical. In publicly funded healthcare systems, like the UK's NHS, funding for TCAM is “*under consistent instability and insecurity*”. TCAM services are expected to be cost-neutral, even if they do not actually bring cost savings to the NHS. Thus, many TCAM services operate on a self-financing basis, and even when public funding is available, caps on the maximum number of treatments are often applied [29], [36], [48]. Under such funding arrangements, the flexibility of TCAM provision may be compromised because services would need to be geared to serving the purpose of cost containment and demand management [25], [37]. For example, patients with acute problems may be given preference over those with chronic problems needing osteopathic treatment since effects will be evident in a shorter time span than for those with chronic diseases [29]. The uneven distribution of TCAM funding amongst population groups and locations has made equity in service access difficult to achieve in most situations [21]. Financial barriers to access have also been reported in health systems with social insurance [22], [30], [43], [47] and private fee for service [28], [34] payment mechanisms. In these health systems, the patients' ability to pay becomes a major determinant in referral rates between BMD and TCMP [19]. Nevertheless, some administrators believe that payment increases patient's power in negotiating how TCAM is provided within health systems [25], [49].

#### Leadership

Leadership can be categorized either as emergent or as related to a position [11]. Acceptance of emergent leadership to integrate services is more likely because it is a shared process with agreement of different partners [21]. BMD are often the emergent leaders because their professional affinity and credibility can better facilitate the innovation of new services [28], [41], [42]. Clinicians accredited both in BM and TCAM (dual accredited clinicians) are particularly suitable as they can anticipate tensions between the two paradigms, and possibly resolve them by translating TCAM terminology into BM terms [31], [39]. The core function of a leader in these circumstances is to seek endorsement and support from stakeholders at various levels of the BM hierarchy [45], [50].

Leaders are expected to have a “*dedication to integration on an organizational level*” [46]. And this can be demonstrated by their efforts in securing approval, funding, space and information systems for the program [28], [50]. The choice of a service site is often of paramount strategic importance. Compared to an acute inpatient environment, primary and palliative care settings are more flexible and are more likely to endorse the provision of TCAM services [27], [42].

At the operational level, leaders need to enlist support from BMD, including those who are not involved in IHC services; this is because peer pressure exerted by them can hinder participation by other interested BMD [34], [42]. Ensuring the safety of additional TCAM treatment is at the heart of the business [39]. Leaders may experience less resistance from the management if the participating TCAMP are accredited in their own discipline, covered by liability insurance, and are willing to stay within their defined scope of practice such that liability concerns would be minimized [19], [28], [30], [44], [48], [50]–[52]. In countries with weak or no TCAMP accreditation systems, or when the TCAM modality is not popular amongst local populations, IPC is less likely to be successful [22], [34], [37], [53]. Preferably, candidates are allied health professionals trained in TCAM [20], [23], [27], or TCAMP who possess some BM credentials. This is because they are more likely to make timely referrals when the patients' conditions fall outside his or her scope of practice [27], [42], [51].

#### Support for innovation

The launching of an IPC program almost always entails the innovative rearrangement of responsibilities, and this innovation cannot take place without organized complementary learning between the partners [11]. Before starting a fully-fledged IHC service, a run-in period that allows familiarization between BMD and TCAM can be beneficial [45], [46], [48], [52]. Bridge-building activities, including mutual practice observation [48] and the creation of communication platforms [52], facilitate bonding development amongst team members [29], [32], [52]. For example, one study reported that integration began with allowing TCAMP to provide opinions on possible treatments, and therapies were not provided until a later stage [50]. Last but not least, interprofessional education between BMD and TCAMP is critical for mutual referral and teamwork [29], [31], [38], [41], [44]. For BMD, lack of knowledge is one of the most cited reasons for disapproving of TCAM [30], [34], [35]. Patient referral to TCAMP is very unlikely unless BMD are willing to gain a better understanding of TCAM, and to incorporate such learning into clinical decision-making [19], [24], [49]. Thus, training should be set at a level where BMD possess sufficient knowledge to work with TCAMP, as full professional training is often impractical [28], [45], [54]. Similar educational approaches can follow for BM training of TCAMP [19], [31], [38], [54].

Ideally, IPC should not be confined only to referral as optimal treatment plans cannot be devised without team communication processes [19], [28], [33], [41]. Although mutual respect is considered by both BMD and TCAMP as being critical for maintaining a smooth collaborative relationship [4], [38], [41], [46], [50], BMD often remain to be the “*orchestra conductors*” and serve as gate-keepers for TCAM service access [19], [22], [28], [38], [45]. This allows BMD to exercise control over TCAMP's scope of practice [54]; this is because TCAMP are often asked to treat specific symptoms that are not responding well to BM approaches [23], [33]. Subordination of TCAM into a paramedical status appears to be a common way for gaining recognition and legitimacy within many BM settings [23], [35], [41], [50], and it is not surprising that some TCAMP may regard this as a major limitation of IPC [19], [23], [27], [30], [41]. Subordination may be perceived as a means to absorb TCAM modalities into the BM tool box, and in the longer term this may sideline the role of TCAMP [4], [53], [54]. In response, they may establish a defensive professional boundary by developing their own body of “*esoteric knowledge*”, as well as absorbing BM knowledge into their practice [44], [54].

Nevertheless, other TCAMP express positive views on this triage function. This is regarded as a “*key to creating the level of comfort necessary to initiate*” TCAM services within BM settings [24], [26], [40], [50]. To ensure safety [31], [35], most TCAMP would agree that there is a clear role for BMD in: (1) screening out patients with life-threatening illnesses; (2) screening out patients with conditions that are treatable with a clear and effective BM therapy; (3) screening out complications of existing disease and the emergence of new diseases; and (4) identifying the need for changes in current BM medications [26], [41], [51]. This gate-keeping function may encourage the use of TCAM as this implies that the referring BMD still holds overall clinical responsibility for patients [30], [33]. Leaders need to reduce the power differential between BMD and TCAMP [19], [38], [42], so that TCAMP can be sufficiently empowered for meaningful participation [44], [47], [49]. To this end, leaders should enroll BMD who have strong personal interests and open attitudes towards TCAM [19], [22], [34], [40], [41], [45], [49]. On the other hand, leaders can motivate TCAMP by emphasizing the benefits of working in a BM environment. These include access to BM facilities and diagnostic tests, wider recognition and higher professional status, stable referral of patients from BMD and increased income [20], [27], [30], [32], [37], [39], [54]. In the longer term, leaders should promote team stability to ensure that there is a higher degree of mutual acquaintance; this will promote teamwork between BMD and TCAMP [19], [45].

#### Connectivity

Connectively refers to “*the fact that individuals and organizations are interconnected, that there are places for discussion and for constructing bonds between them*” [11]. For IHC, the performance of the BMD–TCAMP team depends on the strength of connectivity. This is because effective communication between members allows for rapid and continuous adjustments in response to problems of coordination. The paradigm differences between the two types of clinicians may pose a unique “*language barrier*” that hinders effective communication [22], [30], [34], [36], [41], [45], [51], [54]. Interestingly, for BMD, the perceived degree of foreignness can vary according to the nature of the TCAM intervention. For example, many BMD find acupuncture more acceptable than homeopathy, as the former “*has a clear physiology, diagnosis, pathology, and treatment*”, but the latter does not [25], [29], [35], [39], [42]. A lower degree of perceived foreignness, TCAMP's ability in communicating with common BM terminology, and facilitation by dual trained clinicians can enhance connectedness between the two groups [19], [30]. The need for facilitated communication mandates frequent face-to-face interaction between BMD and TCAM, and it appears that chart-sharing alone would be insufficient for meaningful shared decision-making [47]. Echoing the acknowledged need for strong connectivity between BMD and TCAMP, an evaluation of IHC programs suggested that co-location of BM and TCAM practice is a key to successful integration [28], [47], [50].

The benefits of co-location can be observed both at clinician and operational levels. At clinician level, co-location is critical for fostering trust, as well as developing a sense of partnership between BMD and TCAM [19], [29], [33], [34]. At an operational level, co-location facilitates efficient referral, feedback, communication, chart-sharing and access to BM testing facilities [19], [31], [38], [53]. However, these benefits should be seen as being potential rather than guaranteed; this is because other factors may influence the collaboration process [29]. Firstly, finding space to host TCAM services within BM settings is often difficult [35], [44], [50]. Secondly, co-location alone is insufficient for building up stronger links. BMD and TCAMP may have little interaction despite the fact that they are working under the same roof, as the former may have irregular shifts and the latter may work only part-time in the clinic [19], [33], [47]. Dedicated time for face-to-face communication is considered to be critical, but time constraint appears to be a constant challenge for both sides [19], [22], [29], [40], [41], [45]–[47,47]. A possible solution to this is to make formal meetings part of a continuing education program that will allow participants to earn credits for annual credentialing requirements [30]. Lastly, some TCAMP may perceive co-location as being a potential threat to their autonomy and that it may limit the range of therapies that can be offered [33], [46]. Proponents of separation also suggest that a stand-alone TCAM center can receive referrals from many geographically proximate BM practices, and thus improve the sustainability of the service [37]. In addition, separation can avoid direct conflict between BMD and TCAMP, thereby sparing leaders and mangers from resolving an “epistemological crash” between the two paradigms [42].

### Formalization

Formalization is defined as “*the extent to which documented procedures that communicate desired outputs and behaviours exist and are being used*” [55]. It is an important means of “*clarifying the various partners' responsibilities and negotiating how responsibilities are shared*” [11]. The formalization of IHC service would require structuring care via tools that specify a patient's clinical pathway, as well as timely information exchange between BMD and TCAMP.

#### Formalization Tool

In the context of interprofessional collaboration, formalization tools are often consensus statements that clarify the respective responsibilities of BMD and TCAM, particularly regarding how clinical care responsibility is being delegated from BMD to TCAMP. The included literature highlighted three common types of delegation mechanism: (i) case-by-case referral; (ii) flexible protocol-based referral; and (iii) condition-specific referral protocol.

The first scenario resembles the traditional relationship between BMD and allied health professionals, in which there would be no referral protocol and BMD may refer after an informal consultation with TCAMP. TCAMP may refer the patient back to BM care if they feel that the patient requires treatment beyond their scope of practice [33], [34], [45]. In the second scenario, a protocol would be developed to delineate the responsibilities of BMD and TCAMP, as well as the clinical pathways for patients. However, it serves only as general guidance for clinicians, and the collaboration process would remain flexible. This approach may balance the needs for individualizing the integration treatment for patients on the one hand, and for preventing conflicts between BMD and TCAM on the other [46]. In these two delegation mechanisms, the efficacy of the chosen TCAM therapy is usually not assessed. Instead, managers and BMD's opinions and perceptions on that therapy's safety [24], [48], as well as cost and local availability [25], [42], dominate the decision-making process. The need for replacing “*fruitless epistemological debate*” with smooth collaboration is one possible explanation for their non-adherence to the evidence-based medicine principle, and this could be a reflection of their unannounced acceptance of TCAM [20], [26], [27].

Lack of evidence on the efficacy and safety of TCAM is a common rhetorical tool against integration amongst BMD [21], [36], [40]. In order to avoid opposition, the third form of formalization takes a more conservative approach, in which a specific TCAM treatment protocol is developed for a specific condition [42]. Nevertheless, because this approach would require clinicians to perform the burdensome task of searching and digesting literature on TCAM [25], [51], the potential resistance to its implementation should not be underestimated [22], [38], [44]. Furthermore, for many TCAM modalities, the absence of quality clinical evidence is not uncommon. Under these circumstances, leaders may brand TCAM inclusion as a means for clinical research [27], [28], [35], [36], [50]. This approach is likely to satisfy skeptics because it equates to making TCAM evidence-based [35], [36], [39], [42]. Some TCAMP may also support the initiative as they perceive research as a means to improve personal research competency as well as the creditability of TCAM [36], [41], [44]. However, clinicians often lack formal research training and thus the launch of a successful research program in integration settings would often require extra support from experienced researchers [23], [28], [37], [41], [44]. Some TCAMP may perceive that there is no need to evaluate their practice because the patients' outcomes can speak for themselves, and their treatment effect cannot be captured by conventional research methodology [23], [36], [37], [42].

#### Information Exchange

In IPC, professionals depend on timely information exchange to reduce the uncertainty in their relationships with unfamiliar collaborators. Verbal and written feedback provides professionals with a channel to evaluate the quality of their partners [11]. Chart-sharing, preferably in electronic form, is considered to be a major channel for communication, but legal barriers may exist for such sharing [26], [30], [34]. In integration settings, positive feedbacks from outcome evaluation serve an essential role in building trust between BMD and TCAMP [26], [48]. TCAMP would expect timely feedback from their BMD partner in order to feel respected [53]. This is even more important for BMD because they often fill their knowledge gaps in TCAM by comments from patients and colleagues [33], [36], [45]. Repeated positive feedback may encourage BMD to refer patients who have an increasingly wide range of problems, but they may also become reluctant to refer if feedback was negative for the first referral [30], [33]. For some BMD, such immediate feedback partly replaces the need for clinical trial evidence [29], [36] and it can promote referral decisions [24] as well as create a stronger trust amongst TCAMP [32], [50]. TCAMP may informally make use of this mechanism to gain credibility [44], but more mature integration programs usually formalize their evaluation process around outcomes that are recognized both by BMD and TCAMP [28], [40], [41].

## Discussion

### Integration of BM and TCAM: Lessons from IPC between BMD and Allied Health Professionals

This systematic review is one of the first attempts to summarize how organizational strategies may foster IPC between BMD and TCAMP, as well as their determinants of success and failure. Whereas the integration of BM and TCAM poses a unique challenge to policy-makers and managers at the epistemological level, themes from our synthesis appear to echo the general findings from integration experience amongst other healthcare professionals. Conflicts around role boundaries, the scope of practice, as well as confusion about accountability between BMD and allied health professionals are not uncommon in primary care settings [56]. From a cognitive and psychosocial perspective, Mitchell *et al.* suggested that semantic misunderstanding, lack of cognate mental models, and threats to professional identity are general and persistent barriers in many diversification initiatives for healthcare teams [57]. These barriers are unlikely to be easily removed when power differentials between partners persist. Given the hierarchical power structure within healthcare in which BMD often hold institutional sanction in negotiating how IPC should be implemented, working relationships are often controlled by BMD [58]. Managers without clinical responsibility could be instrumental in effecting coordination and balancing power differentials; as they can devote more energy and time into developing and implementing conflict resolution [56], [59]. However, other studies on IPC leadership suggested that in-depth knowledge in both partners' disciplines is a prerequisite for the successful resolution of interprofessional conflicts [60]. In our synthesis, protocol-based referral agreements between BMD and TCAMP could be regarded as a type of “conflict resolution protocol”, but the development of such referral guidelines is unlikely to be accepted unless the leader understands the perspectives of both BMD and TCAMP. This provides a potential explanation of why integration is often led by dual trained clinicians.

Our review also demonstrated that allied health professionals with TCAM training, or TCAMP with substantial knowledge of BM, would fit better into integration initiatives. Their BM background could help to minimize the negative effect of social categorization when they interact with BMD. This may also explain why TCAMP with more formal employment terms within BM settings appeared to be more willing to communicate well with their partners [57]. Whereas recategorization towards a dominant group identity may improve TCAMP's credibility and their intention to collaborate, the accompanying risk of forgoing TCAM's epistemological stance cannot be overlooked [61]. Recent literature has viewed IPC as a change process, which posits that “no single agent had full authority, resources, or expertise to lead the change” required for implementing IPC in healthcare [59]. Under this perspective, even leaders who are dual-trained both in BM and TCAM would not succeed unless a broad coalition of actors, including TCAMP, were being mobilized to activate change in healthcare delivery. Power sharing in this “distributed agency” approach could be a way to build a more balanced IPC model, in which TCAM leaders could possess more negotiating power in protecting TCAM's practice philosophy. We found that no included studies described this approach, and further investigation on its feasibility should be assessed.

### Strengths and Weaknesses of this Study

In this review we strived to ensure methodological rigor by following previously applied methods for systematic reviews of qualitative studies. With a three-stage systematic approach, comprising extensive literature search, methodological quality assessment, and framework analysis, we synthesized 37 methodologically sound qualitative studies on how organizational factors influence IPC between BMD and TCAMP. As many of the themes emerged in this review have appeared repeatedly across a wide range of heterogeneous studies on various types of TCAM, and the quality of included studies were acceptable, the current synthesized findings may be considered as trustworthy. However, as our results are drawn from reports of the original authors instead of analyzing the raw qualitative data, the conclusions made in this review should be interpreted with caution.

There are several methodological limitations in conducting this systematic review. First, we found it difficult to formulate appropriate search terms for each database. This is due to a lack of consensus on which modality should be regarded as a core therapy under the umbrella of TCAM [62]. We attempted to minimize incomprehensiveness of search by using best available search terms for TCAM. For instance, we have used a very comprehensive MEDLINE search strategy developed by the Cochrane CAM field (http://www.compmed.umm.edu/cochrane_ovid.asp), which is embedded in the “complementary medicine” limit filter in the OVID interface. For other databases which a Cochrane CAM search filter is yet to be developed, we have attempted to maximize sensitivity of search by ensuring the inclusion of generic terms like “complementary therapies” or “alternative medicine” in all search strategies, and make references to search terms published in related systematic reviews [63]. Furthermore, given the experience in TCAM health services research amongst the authors, we were able to sample targeted journals that have a good chance of containing eligible studies. The strategy is found to be effective in searching qualitative research on barriers and facilitators of program delivery [64].

Second, as the exclusion of low quality studies in systematic reviews of qualitative research is controversial [65], we have chosen a more inclusive approach by not excluding any studies based on methodological quality. We attempted to assess the impact of including studies of lower methodological quality by evaluating whether they contribute uniquely to our final synthesis. For the two included studies which satisfied less than 6 quality criteria [29], [52], one study [29] added two unique descriptions to the theme and the other [52] contributed one. This implies that while their inclusion may not cause significant changes in the present synthesis, their exclusion may lead to some loss of descriptive details. Overall speaking, as we have produced findings from a relatively large number of studies, the mediocrity of some included studies may be compensated. The trustworthiness of our synthesis would benefit from further verification in future studies.

Third, we faced challenges in choosing an appropriate analysis approach in our synthesis. It is possible that we would have taken a meta-ethnographical approach, in which first, second and third order constructs would be explored in a more inductive fashion [14]. However, we have decided to choose a more deductive framework approach as it offers practical benefits in focusing our analysis on IPC, a core issue of concerns amongst all stakeholders in IHC settings. In fact, it is a recommended analysis approach when a qualitative synthesis attempts to answer questions relating to health system policies and implementation [66], [67].

### Generalizability of findings and future research needs

Organizational strategies discussed in this synthesis should be interpreted under the context of wider TCAM policies, payment mechanisms [68], as well as cultural differences [69]. For instance, our findings on interprofessional collaboration are not applicable to China, in which both Chinese and BM trained doctors are allowed to prescribe both western and Chinese medications [70]. Also, as the Chinese government has provided strong administrative and financial support for integration, difficulties in creating co-located TCAM-BM clinical settings may not be apparent. In fact, majority of hospitals and community health centers are annexed with Chinese medicine departments [71]. Taking into account these contextual differences, we decided to focus on other health systems which integration is less developed. According to a survey published by the World Health Organization in 2005, 96 out of 141 responding member states are yet to develop relevant TCAM policies [72]. Our findings may be useful for policy makers in these health systems and practical strategies yield from this synthesis is listed in Table S3.

Future research would need to assess barriers in implementing these strategies. For instance, leaders of IHC may face ethical challenges in adhering to the principles of beneficence and non-maleficence when BM and TCAM clinicians held different views on the risk-benefit ratio of using an integrative approach [73], [74]. In addition, the applicability of our proposed strategies may vary according to the context where collaborations take place. Stewardship for collaboration in research and routine practice settings maybe require different emphasis.

Finally, given the wide geographical distribution of the studies' origin, careful consideration should be given when policy-makers and managers attempt to generalize our findings. The majority of included studies were conducted in Canada and the UK, in which respectively 70% [75] and 46.6% [76] of the population had ever used TCAM. The paucity of studies from other countries where TCAM is more popular has prevented us from exploring the heterogeneity of views across different locations. Given the differences in socio-cultural contexts, management structures and the training of healthcare professionals in different countries, the appropriateness of generalizing our findings to any specific health system should be judiciously scrutinized, particularly amongst policy makers from outside Canada or the UK.

## Conclusions

This systematic review synthesized 37 qualitative studies that investigated organizational strategies for fostering interprofessional collaboration between BMD and TCAMP. The themes that have emerged from our synthesis suggest that the nature of relationships between BMD and TCAMP are similar to those between BMD and allied health professionals. Therefore, interventions for improving team effectiveness in other healthcare teams with different compositions [77] may well be transferable for improving IPC between BMD and TCAMP. In the longer run, researchers must demonstrate how the addition of TCAMP, and possibly “IHC coordinators”, can improve care quality, reduce costs, and ultimately benefit patients [78]. Finally, future qualitative research in this area should address common methodological shortcomings in the presentation of data analysis method, reflection of researchers' role in the study settings, self appraisal on study quality, and description on future research needs.

## Supporting Information

Appendix S1 **Search Strategy for MEDLINE (OVID, 1950 – Week 1 March 2011).**
(DOCX)

Appendix S2 **Purposive Search of 16 Health Services Journals: Strategy for MEDLINE (OVID, 1950 – Week 1 March 2011).**
(DOCX)

Checklist S1 **PRISMA 2009 Checklist.**
(DOCX)

Table S1 **Characteristics of included studies.**
(DOCX)

Table S2 **Methodological quality of included studies.**
(DOCX)

Table S3 **Summary of themes generated from the synthesis.**
(DOCX)
