# Supplementary material for: Organizational Determinants of Interprofessional Collaboration in Integrative Health Care: Systematic Review of Qualitative Studies
Source: PLoS One. 2012 Nov 29;7(11):e50022. doi: 10.1371/journal.pone.0050022 (PMC3510174; doi:10.1371/journal.pone.0050022)
Supplement: Appendix S1 — Search Strategy for MEDLINE (OVID, 1950 – Week 1 March 2011). (DOCX) [file pone.0050022.s001.docx]

**Appendix S1**

**Search Strategy for MEDLINE (OVID, 1950 – Week 1 March 2011)**

1. exp Interprofessional Relations/ and (collaborat$ or team$).tw. (7283)

2. exp Patient Care Team/ and (collaborat$ or team$).tw. (15380)

3. ((interprofession$ or inter-profession$) adj (collaborat$ or team$)).tw. (399)

4. ((interdisciplin$ or inter-disciplin$) adj (collaborat$ or team$)).tw. (2499)

5. ((interoccupation$ or inter-occupation$) adj (collaborat$ or team$)).tw. (0)

6. ((multiprofession$ or multi-profession$) adj (collaborat$ or team$)).tw. (279)

7. ((multidisciplin$ or multi-disciplin$) adj (collaborat$ or team$)).tw. (6323)

8. ((transdisciplin$ or trans-disciplin$) adj (collaborat$ or team$)).tw. (75)

9. (team$ adj collaborat$).tw. (116)

10. 1 or 2 or 3 or 4 or 5 or 6 or 7 or 8 or 9 (25927)

11. limit 10 to ("qualitative studies (optimized)" and complementary medicine) (3700)

**Search Strategy for EMBASE (OVID, 1980 – Week 1 March 2011)**

1. exp Interprofessional Relations/ and (collaborat$ or team$).tw. (5645)

2. exp Patient Care Team/ and (collaborat$ or team$).tw. (22781)

3. ((interprofession$ or inter-profession$) adj (collaborat$ or team$)).tw. (493)

4. ((interdisciplin$ or inter-disciplin$) adj (collaborat$ or team$)).tw. (3262)

5. ((interoccupation$ or inter-occupation$) adj (collaborat$ or team$)).tw. (1)

6. ((multiprofession$ or multi-profession$) adj (collaborat$ or team$)).tw. (400)

7. ((multidisciplin$ or multi-disciplin$) adj (collaborat$ or team$)).tw. (9071)

8. ((transdisciplin$ or trans-disciplin$) adj (collaborat$ or team$)).tw. (103)

9. (team$ adj collaborat$).tw. (128)

10. 1 or 2 or 3 or 4 or 5 or 6 or 7 or 8 or 9 (35969)

11. limit 10 to "qualitative studies (2 or more terms min difference)" (10275)

12. *alternative medicine/ or *integrative medicine/ or *holistic care/ or *herb/ or *chinese herb/ or *acupuncture/ or *chiropractic practice/ or *chiropractic/ or *manipulative medicine/ or *bodywork/ or *chinese medicine/ or *qi gong/ or *massage/ or *ayurveda/ or *ayurveda drug/ or *traditional medicine/ (51150)

13. 11 and 12 (67)

**Search Strategy for AMED (OVID, 1985 – Week 1 March 2011)**

1. exp Interprofessional Relations/ and (collaborat$ or team$).tw. (373)

2. exp Patient Care Team/ and (collaborat$ or team$).tw. (1476)

3. ((interprofession$ or inter-profession$) adj (collaborat$ or team$)).tw. (39)

4. ((interdisciplin$ or inter-disciplin$) adj (collaborat$ or team$)).tw. (328)

5. ((interoccupation$ or inter-occupation$) adj (collaborat$ or team$)).tw. (0)

6. ((multiprofession$ or multi-profession$) adj (collaborat$ or team$)).tw. (39)

7. ((multidisciplin$ or multi-disciplin$) adj (collaborat$ or team$)).tw. (397)

8. ((transdisciplin$ or trans-disciplin$) adj (collaborat$ or team$)).tw. (13)

9. (team$ adj collaborat$).tw. (20)

10. 1 or 2 or 3 or 4 or 5 or 6 or 7 or 8 or 9 (2174)

11. limit 10 to (acupuncture or complementary medicine or "herbalism or herbal drugs" or "homeopathy or homeopathic drugs" or hypnosis or medicinal plants or traditional medicines) (45)

**Search Strategy for CINAHL (EBSCOHost, 1982 – Week 1 March 2011)**

1. (MH Interprofessional Relations+) (15481)
2. (MH "Multidisciplinary Care Team+") (18581)
3. TX collaborat* OR team* (91447)
4. TX Interprofession* OR inter-profession* (13689)
5. TX Interprofession* OR inter-profession* n1 collaborat* OR team* (61275)
6. TX Interoccupation* OR inter-occupation* (4)
7. TX Interoccupation* OR inter-occupation* n1 collaborat* OR team*  (50805)
8. TX Multiprofession* OR multi-profession* (868)
9. TX Multiprofession* OR multi-profession* n1 collaborat* OR team* (51053)
10. TX Transdisciplin* OR trans-disciplin* (610)
11. TX Transdisciplin* OR trans-disciplin* n1 collaborat* OR team* (51245)
12. TX Multidisciplin* OR multi-disciplin* (28685)
13. TX Multidisciplin* OR multi-disciplin* n1 collaborat* OR team* (57913)
14. TX Multioccupation* OR multi-occupation* (0)
15. TX Multioccupation* OR multi-occupation* n1 collaborat* OR team* (50804)
16. TX Team* n1 collaborat* (512)
17. (1-16)/OR (110268)
18. (MH "Alternative Therapies+") (95517)
19. (MH "Holistic Care") (2165)
20. (MH "Plants, Medicinal+") (30542)
21. (MH "Drugs, Chinese Herbal") or (MH "Medicine, Chinese Traditional+") (10817)
22. (MH "Acupuncture+") or (MH "Acupuncturists") (7233)
23. (MH "Chiropractic+") or (MH "Chiropractic Practice") or (MH "Manipulation, Chiropractic") (15448)
24. (MH "Manual Therapy") (2015)
25. (MH "Manual Therapy+") (23479)
26. (MH "Qigong") (207)
27. (MH "Massage+") or (MH "Swedish Massage") or (MH "Deep Tissue Massage") or (MH "Massage Therapists")  (7450)
28. (MH "Medicine, Ayurvedic") (699)
29. (MH "Medicine, African Traditional") or (MH "Medicine, Latin American Traditional") or (MH "Medicine, Oriental Traditional+") or (MH "Medicine, Traditional+") or (MH "Medicine, Native American") or (MH "Traditional Healers") (19556)
30. (MH "Integrative Medicine") (1773)
31. (18-30)/OR (125330)
32. 17 AND 31 (3187)
33. **Limiters** - Clinical Queries: Qualitative - Best Balance, limit to 31^st^ Mar 2011 (151)
