# Supplementary material for: Organizational Determinants of Interprofessional Collaboration in Integrative Health Care: Systematic Review of Qualitative Studies
Source: PLoS One. 2012 Nov 29;7(11):e50022. doi: 10.1371/journal.pone.0050022 (PMC3510174; doi:10.1371/journal.pone.0050022)
Supplement: Appendix S2 — Purposive Search of 16 Health Services Journals: Strategy for MEDLINE (OVID, 1950 – Week 1 March 2011). (DOCX) [file pone.0050022.s002.docx]

**Appendix S2**

**Purposive Search of 16 Health Services Journals: Strategy for MEDLINE (OVID, 1950 – Week 1 March 2011)**

1. medical care.jn. (5574)

2. health expectations.jn. (370)

3. "british journal of general practice".jn. (5312)

4. social science & medicine.jn. (11097)

5. value in health.jn. (913)

6. medical decision making.jn. (1605)

7. health services research.jn. (2437)

8. evaluation & the health professions.jn. (686)

9. "medical care research & review".jn. (532)

10. qualitative health research.jn. (1253)

11. "journal of interprofessional care".jn. (605)

12. "sociology of health & illness".jn. (534)

13. bmc health services research.jn. (1326)

14. "journal of ambulatory care management".jn. (1204)

15. family practice.jn. (2338)

16. "annals of family medicine".jn. (835)

17. 1 or 2 or 3 or 4 or 5 or 6 or 7 or 8 or 9 or 10 or 11 or 12 or 13 or 14 or 15 or 16 (36621)

18. limit 17 to (abstracts and "qualitative studies (optimized)" and complementary medicine) (5023)

19. from 18 keep 1-5023 (5023)

**Purposive Search of 15 CAM Journals: Strategy for EMBASE and MEDLINE (OVID, 1950 – Week 1 March 2011)**

Database: EMBASE

Search Strategy:

--------------------------------------------------------------------------------

1. "evidence based complementary and alternative medicine".jn. (614)

2. ("acupuncture and electro therapeutics research" or acupuncture in medicine).jn. (924)

3. "explore the journal of science and healing".jn. (548)

4. homeopathy.jn. (427)

5. "american journal of chinese medicine".jn. (1034)

6. "journal of manipulative and physiological therapeutics".jn. (2814)

7. (forschende komplementarmedizin or forschende komplementarmedizin 2006 or forschende komplementarmedizin und klassische naturheilkunde).jn. (810)

8. altex alternativen zu tierexperimenten.jn. (245)

9. "alternative medicine review".jn. (430)

10. "alternative therapies in health and medicine".jn. (1357)

11. integrative cancer therapies.jn. (440)

12. complementary therapies in medicine.jn. (819)

13. 1 or 2 or 3 or 4 or 5 or 6 or 7 or 8 or 9 or 10 or 11 or 12 (10462)

14. limit 13 to "qualitative studies (2 or more terms min difference)" (1421)

Database: MEDLINE

Search Strategy:

--------------------------------------------------------------------------------

1. "journal of alternative & complementary medicine".jn. (1949)

2. complementary therapies in clinical practice.jn. (233)

3. complementary therapies in nursing & midwifery.jn. (364)

4. 3 or 1 or 2 (2546)

5. limit 4 to "qualitative studies (optimized)" (412)

6. from 5 keep 1-412 (412)
