# Supplementary material for: Organizational Determinants of Interprofessional Collaboration in Integrative Health Care: Systematic Review of Qualitative Studies
Source: PLoS One. 2012 Nov 29;7(11):e50022. doi: 10.1371/journal.pone.0050022 (PMC3510174; doi:10.1371/journal.pone.0050022)
Supplement: Table S1 — Characteristics of included studies. (DOCX) [file pone.0050022.s004.docx]

**Table S1 Characteristics of included studies**

| **Source:** Citation #, first author and publication year | **Country and year** | **Participants** | | | | | | **Response rate (%)** | **Sample origin** | **Data collection** | **Methodology** | **Analysis** |
| --- | --- | --- | --- | --- | --- | --- | --- | --- | --- | --- | --- | --- |
|  |  | BMD | TCAMP | Manager | Allied health professional | Patient | Total |  |  |  |  |  |
| 34. Allareddy 2007 | USA 2002-4 | √ (round two focus group n=4) | √ (round two focus group n=13) |  |  |  | Round one focus group: not stated Round two focus group: 17 | Not stated | Academic and community leaders of their respective field | 2-round focus group interviews | Not stated | Content analysis |
| 50. Boon 2008 | Canada 2004-2007 | √ | √ | √ | √ |  | 42 * | Not stated | Two integrative medicine programs within hospitals | Applied ethnography including in depth Interviews and observation of team meetings, retreats, inter-professional interactions and clinic operation | Ethnography | Content analysis |
| 24. Brien 2008 | UK, 2006-2007 | √ (n=10) |  |  |  |  | 10 | 30.3% | Rural and deprived urban GP practices within the NHS | Semi-structured interviews with GP who have referred patients to the private TCAM sector in the past 2 years | Not stated | Framework analysis |
| 42. Broom 2007 | UK, year not reported | √ (n=12) | √ (n=6) |  | √ (n=13) |  | 31 | Not stated | One hospital and one hospice where TCAM treatments were provided | In-depth interviews | Charmaz’s approach to social analysis | Not stated |
| 33. Emanual 1999 | UK, year not reported | √ (n=8) | √ (1^st^ interview n=7) (2^nd^ interview n=5) |  |  |  | 15 | (100% - 1^st^ interview) (86.7% - 2^nd^ interview) | A practice network were BMD employ TCAMP to provide services | Semi-structured interviews | Not stated | Not stated |
| 51. Frenkel 2003 | Israel and USA, year not reported | √ | √ |  |  |  | Not stated | Not stated | Not specified | Multiple methods including literature reviews, key informant interviews, focus groups, education presentations and field testing. | Not stated | Immersion/crystallization analysis |
| 19. Gaboury 2009 | Canada, 2007 | √ ( n=7) | √ (n=11) |  | √ (n=3) |  | 21 | 95.5% | Five urban, “free standing” integrative health care clinics | In-depth, semi structured interviews | Not stated | Content analysis |
| 32. Garner 2008 | Canada 2004-2006, | √ (n=6) |  |  | √ (n=6) |  | 12 | 60% | Two community health centers | Mixed method design with qualitative and quantitative components, using questionnaires and focus group | Not stated | Post hoc analysis, Framework analysis |
| 31. Grace 2008 | Australia, year not reported | √ (n=10) | √ (n=13) |  |  |  | 23 | 92% | 16 integrative practices and one non-integrative general medical practice | In depth interviews | Grounded theory | Constant comparative analysis |
| 52. Haahr 2006 | Denmark, 2002 | √ |  |  | √ |  | 8* | Not stated | A multiple sclerosis specialist hospital in Denmark | Semi-structured interviews. | Not stated | Not stated |
| 44. Halpin 2006 | UK, year not reported |  | √ (n=4) |  |  |  | 4 | Not stated | TCAM services coordinator training workshop | Focus group interview | Not stated | Constant comparative analysis |
| 54. Hollenberg 2006 | Canada, 2002-2003 | √ (n=13) | √ (n=8) |  |  |  | 21 | Not stated | An independent and free-standing integrative health care (IHC) clinic, and an IHC clinic in a hospital | Case study including in-depth semi-structured interviews, ethnographic observation, document analysis | Ethnography | Comparative analysis |
| 43. Hollenberg 2007 | Canada, 2002-2003 | √ (n=15) | √ (n=8) | √ (n=2) |  | √ (n=13) | 38 | Not stated | Three integrative healthcare centre (IHC) settings in Canada | Semi-structured interviews, ethnographic observation, document analysis | Ethnography | Comparative analysis |
| 38. Hsiao 2006 | USA, 2003 | √ (n=26) | √ (n=24) |  |  |  | 50 | Not stated | Private practice and academic medical centres in Los Angeles | Semi-structured, in-depth interviews | Grounded theory | Exploratory technique |
| 49. Paterson 1995 | UK, 1993-1994 | √ (n=5) | √ (n=9) | √ (n=10) | √ (n=7) |  | 31 | Not stated | A four-partner, non-fund holding urban practice including both BMD and TCAMP | Cooperative inquiry (action research) and semi-structured interviews | Not stated | Not stated |
| 29. Reason 1995 | UK, year not reported | √ | √ |  |  |  | 6* | Not stated | A NHS general practice | Tape recorded conversation with both WMD and TCAMP | Not stated | Not stated |
| 23. Theberge 2007 | Canada, year not reported | √ (n=11) | √ (n=8) |  | √ (n=16) |  | 35 | Not stated | A team which worked with top-level athletes and at major international competitions including in most cases the Olympic Games | In-depth interviews | Not stated | Not stated |
| 21. Thomas 2003 | UK, October 1999-February 2001 | √ |  | √ (n=9) |  |  | Not stated | Not stated | NHS primary care environment | Semi structured telephone interviews | Not stated | Framework analysis |
| 41. Launsø 2007 | Denmark August 2004-May 2005 | √ | √ |  | √ |  | 10 * | Not stated | Specialised hospital for multiple sclerosis | Semi-structured in-depth interviews | Not stated | Not stated |
| 37. Luff 2000 | UK, year not reported | √ | √ |  |  |  | 60 * | Not stated | 10 existing schemes of complementary therapy provision in primary care across England | Case study including semi-structured interviews, observation and review of records | Not stated | Framework analysis |
| 39. Mizrachi 2005 | Israel, 2001. | √ | √ | √ |  |  | Not stated | Not stated | Internal medicine department of a hospital | Observations, formal and informal interviews, In-depth interviews, | Not stated | Not stated |
| 35. Mizrachi 2005 | Israel, 1999-2001. | √ (n=12) | √ (n=17) (7 alternative practitioners who were also biomedically trained) |  |  |  | 29 | 90.6% | Ambulatory clinics where alternative care is practiced, and in hospitals where alternative care is provided | In depth interviews supplemented by analysis of two formal policy documents and participant observations | Not stated | Content analysis |
| 46. Mulkins 2005 | Canada, 2003 | √ | √ |  | √ |  | 16 * | Not stated | Tzu Chi Institution (TCI) | In depth semi-structured interviews | Not stated | Not stated |
| 20. Shuval 2002 | Israel, 2000 | √ (n=6) | √ (n=10) |  | √ (n=3) |  | 19 | Not stated | Four Jewish sponsored general hospitals | Semi-structured interviews and observation | Not stated | Not stated |
| 27. Shuval 2004 | Israel, 1999-2001 | √ (n=3) | √ (n=23) (13 alternative practitioners who were also biomedically trained) |  | √ (n=3) |  | 29 | *Clinics:* 82.4% *Hospitals:* Not stated | Four alternative ambulatory clinics and four Jerusalem hospitals | semi-structured in-depth interviews, observation and review of written documents | Not stated | Not stated |
| 47. Soklaridis 2009 | Canada, year of data collection not reported | √ (Total BM and CAM n=10) | √ | *√* (n=13) |  | √ (n=8) | 31 (*Individual interview:* n=10, *Focus group*: n=26) | Not stated | IM health clinic within a university hospital | In-depth interviews and semi-structured focus group | Not stated | Constant comparative analysis |
| 22. Sundberg 2007 | Sweden 2003-2006 | √ | √ | √ |  |  | Not stated | Not stated | Unit for Studies of Integrative Health Care at Karolinska Institute | Notes from research group and key informant meetings, individual field notes from seminars and meetings, written material | Not stated | Immersion and crystallizationResearch group consensus |
| 28. Vohra 2005 | North America, 2002-2003 |  |  | √ |  |  | Not stated | Not stated | 9 leading north American integrative medical centres | Face-to-face interview | Not stated | Content analysis |
| 48. Wye 2008 | UK, 2004-2006 | √ (n=9) |  | √ (n=7) | √ (n=4) |  | 20 | 95.2% | 2 NHS sites offering TCAM services | Semi-structured interview | Not stated | Descriptive content analysis |
| 25. Wye 2009 | UK, year of data collection not reported | √ |  | √ | √ |  | 18* | 100% | 2 NHS sites offering TCAM services | Face-to-face interview, telephone interview, observation and document review | Not stated | Constant comparative analysis |
| 36. Beattie 2010 | UK, year of data collection not reported | √ (n=5) | √ (n=5) |  | √ (n=10) |  | 20 | Not stated | General practices and complimentary practice settings within the Bristol area | Semi-structured in-depth interview | Not stated | Constant comparison analysis |
| 26. Grace 2010 | Australia, year of data collection not reported | √* | √* |  |  | √* | Not stated | Not stated | Several Australian integrative medicine clinics where general medical practitioner and TCAM practitioners were co-located | Cumulative case studies, focus groups, key information interviews | Hermeneutic phenomenology method | Constant comparison analysis |
| 40. Isabelle Gaboury, 2010 | Canada, January and March 2007 | √ (n=9) | √ (n=12) |  | √ |  | 21 | 95.5% | Canadian integrative healthcare (IHC) clinics | Face-to-face interview with semi-structured open-ended questionnaires | Not stated | Content analysis |
| 30. Silvano Mior, 2010 | Canada, year of data collection not reported | √ (individual interview n=4Focus group n=11) | √ (individual interview n=5 focus group n=17) | √ (n=4) (individual interview:academia n=3; Administrationn=1) | √ (individual interview: midwifery n=1, nursing n=3, physiotherapy n=1) | √ (focus group n=34) | Individual interview n=16Focus group: n=62 | Not stated | Two primary care networks (PCN) sites in Ontario, Canada | Key informant interviews and Focus group | Grounded theory | Constant comparison analysis |
| 6. Heather S. Boon, 2009 | Canada, September and December 2001 | √ (n=4) | √ (n=5) | √ (n=3) | √ (n=4) |  | 16 | 94.1% | Primary healthcare setting | Semi-structured interview | Grounded theory | Constant comparison analysis |
| 53. Campbell-Hall, 2010 | South Africa, February 2007 - November 2007 | √ | √ |  | √ | √ (n=15) | Not stated | Not stated | Formal health sector as well as in NGO settings | Individual interview and focus group | Not stated | Framework analysis |
| 45. Lasse Skovgaard, 2010 | Denmark 2004-2010 | √ (n=1) | √ (n=5) |  | √ (n=4) | √ (n=59) | Not stated | Not stated | Danish Multiple Sclerosis (MS) hospital | Individual and group interview | Not stated | Not stated |

Key: BMD: Biomedical Doctors, GP: General Practitioners, NH: UK National Health Service, TCAMP: Traditional, complementary and alternative medicine practitioner * Break down of numbers not reported
