# Supplementary material for: Organizational Determinants of Interprofessional Collaboration in Integrative Health Care: Systematic Review of Qualitative Studies
Source: PLoS One. 2012 Nov 29;7(11):e50022. doi: 10.1371/journal.pone.0050022 (PMC3510174; doi:10.1371/journal.pone.0050022)
Supplement: Table S2 — Methodological quality of included studies. (DOCX) [file pone.0050022.s005.docx]

**Table S2: Methodological quality of included studies**

| Citation #, first author and publication year | **Introduction** | | **Methods** | | | | | **Results** | | **Discussion** | | **Conclusion** |
| --- | --- | --- | --- | --- | --- | --- | --- | --- | --- | --- | --- | --- |
|  | The issue is described clearly and corresponds to the current state of knowledge | The research question and objectives are clearly stated and are relevant to qualitative research | The context of the study and the researcher’s roles are clearly described | The method is appropriate for the research questions | The selections of participants is appropriate to the research question and to the method selected | The process for collecting data is clear and relevant | Data analysis is credible | The main results are presented clearly | The quotations make it easier to understand the results | The results are interpreted in credible and innovative ways | The limitations of the study are presented | The conclusions presents a synthesis of the study and proposes avenues for further research |
| 34. Allareddy 2007 | Unclear | √ | Unclear | √ | √ | √ | √ | √ | √ | √ | √ | √ |
| 50. Boon 2008 | √ | √ | Unclear | √ | √ | √ | √ | √ | √ | √ | √ | √ |
| 24. Brien 2008 | Unclear | √ | Unclear | √ | √ | √ | √ | √ | √ | √ | √ | √ |
| 42. Broom 2007 | √ | √ | Unclear | √ | √ | √ | √ | √ | √ | √ | Not reported | Not reported |
| 33. Emanuel 1999 | Unclear | √ | Unclear | √ | √ | √ | Uncertain | √ | √ | √ | Not reported | Not reported |
| 51. Frenkel 2003 | √ | √ | Unclear | √ | √ | √ | √ | √ | Not reported | √ | Not reported | Not reported |
| 19. Gaboury 2009 | √ | √ | Unclear | √ | √ | √ | √ | √ | √ | √ | √ | √ |
| 32. Garner 2008 | Unclear | √ | Unclear | √ | √ | √ | Uncertain | √ | √ | √ | √ | Not reported |
| 31. Grace 2008 | Unclear | √ | Unclear | √ | √ | √ | √ | √ | √ | √ | Not reported | Not reported |
| 52. Haahr 2006 | √ | √ | Unclear | √ | √ | Unclear | Uncertain | √ | Not reported | Not reported | Not reported | Not reported |
| 44. Halpin 2006 | Unclear | √ | Unclear | √ | √ | √ | Uncertain | √ | √ | √ | √ | √ |
| 54. Hollenberg 2006 | √ | √ | Unclear | √ | √ | √ | Uncertain | √ | √ | √ | Not reported | Not reported |
| 43. Hollenberg 2007 | √ | √ | Unclear | √ | √ | √ | Uncertain | √ | √ | √ | Not reported | Not reported |
| 38. Hsiao 2006 | Unclear | √ | Unclear | √ | √ | √ | √ | √ | √ | √ | √ | Not reported |
| 49. Paterson 1995 | Unclear | √ | Unclear | √ | √ | Unclear | Uncertain | √ | Not reported | √ | √ | Not reported |
| 29. Reason 1995 | Unclear | √ | Unclear | √ | √ | Unclear | Uncertain | √ | √ | Not reported | Not reported | Not reported |
| 23. Theberge 2007 | √ | √ | Unclear | √ | √ | √ | Uncertain | √ | √ | √ | Not reported | Not reported |
| 21. Thomas 2003 | Unclear | √ | Unclear | √ | √ | √ | Uncertain | √ | √ | Not reported | Not reported | Not reported |
| 41. Launsø 2007 | √ | √ | Unclear | √ | √ | √ | √ | √ | √ | √ | Not reported | Not reported |
| 37. Luff 2000 | Unclear | √ | Unclear | √ | √ | √ | Uncertain | √ | √ | √ | Not reported | √ |
| 39. Mizrachi 2005 | √ | √ | Unclear | √ | √ | √ | Uncertain | √ | √ | √ | Not reported | Not reported |
| 35. Mizrachi 2005 | √ | √ | Unclear | √ | √ | √ | Uncertain | √ | √ | √ | Not reported | Not reported |
| 46. Mulkins 2005 | Unclear | √ | Unclear | √ | √ | √ | Uncertain | √ | √ | √ | Not reported | Not reported |
| 20. Shuval 2002 | √ | √ | Unclear | √ | √ | √ | Uncertain | √ | √ | √ | Not reported | Not reported |
| 27. Shuval 2004 | √ | √ | Unclear | √ | √ | √ | Uncertain | √ | Not reported | √ | Not reported | Not reported |
| 47. Soklaridis 2009 | √ | √ | Unclear | √ | √ | √ | √ | √ | √ | √ | √ | √ |
| 22. Sundberg 2007 | √ | √ | Unclear | √ | √ | √ | Uncertain | Unclear | Not reported | √ | Not reported | Not reported |
| 28. Vohra 2005 | Unclear | √ | Unclear | √ | √ | √ | √ | √ | Not reported | √ | √ | Not reported |
| 48. Wye 2008 | Unclear | √ | Unclear | √ | √ | √ | √ | √ | √ | √ | √ | Not reported |
| 25. Wye 2009 | √ | √ | Unclear | √ | √ | √ | √ | √ | √ | √ | Not reported | Not reported |
| 36. Angela Beattie, 2010 | Unclear | √ | Unclear | √ | √ | √ | √ | √ | √ | √ | √ | Not reported |
| 26. Sandra Grace, 2010 | Unclear | √ | Unclear | √ | √ | √ | √ | √ | √ | √ | Not reported | Not reported |
| 40. Isabelle Gaboury, 2010 | Unclear | √ | Unclear | √ | √ | √ | √ | √ | √ | √ | √ | √ |
| 30. Silvano Mior, 2010 | Unclear | √ | Unclear | √ | √ | √ | √ | √ | √ | √ | √ | Not reported |
| 6. Heather S. Boon, 2009 | √ | √ | Unclear | √ | √ | √ | √ | √ | √ | √ | √ | Not reported |
| 53. Vicky Campbell-Hall, 2010 | √ | √ | Unclear | √ | √ | √ | √ | √ | √ | √ | Not reported | Not reported |
| 45. Lasse Skovgaard, 2010 | Unclear | √ | Unclear | √ | √ | √ | √ | √ | √ | √ | Not reported | Not reported |
