# Supplementary material for: Organizational Determinants of Interprofessional Collaboration in Integrative Health Care: Systematic Review of Qualitative Studies
Source: PLoS One. 2012 Nov 29;7(11):e50022. doi: 10.1371/journal.pone.0050022 (PMC3510174; doi:10.1371/journal.pone.0050022)
Supplement: Table S3 — Summary of themes generated from the synthesis. (DOCX) [file pone.0050022.s006.docx]

**Table S3: Summary of themes generated from the synthesis**

| IPC INDICATORS | KEY POINTS |
| --- | --- |
| 1. Governance | - The need to respond to patients’ demand for holistic care, and the need to fill the effectiveness gaps of BM are the main drivers for integration initiatives. |
|  | - Sufficient funding is essential for building a collaboration-ready TCAMP team and promoting equality for patient access, but securing such funding is a major challenge for central authorities. |
| 2. Leadership | - Clinicians trained both in BM and TCAM are preferred candidates for leading integration. |
|  | - Leaders may experience less resistance from the management if the participating TCAMP are accredited in their own discipline, covered by liability insurance, and are willing to stay within their defined scope of practice. |
| 3. Support for innovation | - Bridge-building activities, including interprofessional education, mutual practice observation and the creation of communication platforms, are beneficial for creating bonding between team members. |
|  | - Gate-keeping of TCAM services by BMD may elicit a mixed response from TCAMP. Leaders need to reduce this power differential such that TCAMP can be sufficiently empowered for meaningful participation. |
| 4. Connectivity | - BMD is better connected with TCAM modalities that have a lower degree of perceived foreignness – *e.g*., acupuncture is more accepted than homeopathy. |
|  | - At clinician level, co-location is critical in fostering trust and also in developing a sense of partnership between BMD and TCAM. |
|  | - At an operational level, co-location facilitates efficient referral, feedback, communication, chart-sharing and access to BM testing facilities. |
| 5. Formalization tools | - Included literature highlighted three common types of delegation mechanisms: (i) case-by-case referral; (ii) flexible protocol-based referral; and (iii) condition-specific referral protocol. |
|  | - In the first two delegation mechanisms, the efficacy of the chosen TCAM therapy is usually not assessed. Instead, managers’ and BMD’s opinions and perceptions on that therapy’s safety, as well as the cost and local availability, dominate the decision-making process. |
|  | - In the third approach, leaders may brand TCAM inclusion as a means for clinical research and this is likely to satisfy skeptics. However, success would often require extra support from experienced researchers. |
| 6. Information exchange | - Shared electronic health record is the preferred mode of information exchange between BMD and TCAMP. |
|  | - Immediate positive feedback received via record exchange can promote BMD’s referral decisions as well as create a stronger trust in TCAMP’s competency. |
|  | - More mature integration programs usually formalize their evaluation process around outcomes that are recognized both by BMD and TCAMP. |
